# Gut mucosa dissociation protocols influence cell type proportions and single-cell gene expression levels

**DOI:** 10.1038/s41598-022-13812-y

**Published:** 2022-06-14

**Authors:** Werna T. C. Uniken Venema, Aarón D. Ramírez-Sánchez, Emilia Bigaeva, Sebo Withoff, Iris Jonkers, Rebecca E. McIntyre, Mennatallah Ghouraba, Tim Raine, Rinse K. Weersma, Lude Franke, Eleonora A. M. Festen, Monique G. P. van der Wijst

**Affiliations:** 1 Department of Gastroenterology and Hepatology, University of Groningen, University Medical Center Groningen, Groningen, The Netherlands; 2 Department of Genetics, University of Groningen, University Medical Center Groningen, Groningen, the Netherlands; 3 Wellcome Sanger Institute, Hinxton, Cambridgeshire, CB10 1SA UK; 4 Department of Gastroenterology, Addenbrooke’s Hospital, Cambridge University Hospitals NHS Foundation Trust, Cambridge, UK

**Keywords:** Genetics research, Transcriptomics

## Abstract

Single-cell RNA sequencing (scRNA-seq) has revolutionized the study of the cellular landscape of organs. Most single-cell protocols require fresh material, which limits sample size per experiment, and consequently, introduces batch effects. This is especially true for samples acquired through complex medical procedures, such as intestinal mucosal biopsies. Moreover, the tissue dissociation procedure required for obtaining single cells is a major source of noise; different dissociation procedures applied to different compartments of the tissue induce artificial gene expression differences between cell subsets. To overcome these challenges, we have developed a one-step dissociation protocol and demonstrated its use on cryopreserved gut mucosal biopsies. Using flow cytometry and scRNA-seq analysis, we compared this one-step dissociation protocol with the current gold standard, two-step collagenase digestion, and an adaptation of a recently published alternative, three-step cold-active *Bacillus licheniformus* protease digestion. Both cell viability and cell type composition were comparable between the one-step and two-step collagenase dissociation, with the former being more time-efficient. The cold protease digestion resulted in equal cell viability, but better preserves the epithelial cell types. Consequently, to analyze the rarer cell types, such as glial cells, larger total biopsy cell numbers are required as input material. The multi-step protocols affected cell types spanning multiple compartments differently. In summary, we show that cryopreserved gut mucosal biopsies can be used to overcome the logistical challenges and batch effects in large scRNA-seq studies. Furthermore, we demonstrate that using cryopreserved biopsies digested using a one-step collagenase protocol enables large-scale scRNA-seq, FACS, organoid generation and intraepithelial lymphocyte expansion.

## Introduction

Even though it has been over 30 years ago that the first cDNA was extracted from a single cell^1^, it is only very recently that technological developments in the field of single-cell RNA-sequencing (scRNA-seq) allow single-cell gene expression profiling in a high throughput manner^2^. This development enables the unbiased study of heterogeneous cell populations within tissues without the need for isolating cell types separately. Beyond mapping cellular expression, this allows studying potential cell–cell interactions within the context of a tissue^3^.

Several worldwide efforts are ongoing to map the human body at single-cell resolution in both health (Human Cell Atlas^4^) and disease (LifeTime consortium^5^). One of the fields in which large efforts are already being undertaken is the field of gastroenterology; fetal, pediatric and adult ‘healthy’ gut atlases are (currently being) generated^6,7^. This has resulted in the identification of new cell types and their specific functions, such as the BEST4 epithelial cells involved in pH sensing^8^, and has provided first clues on why some patients with Crohn’s disease do not respond to specific medication^9^.

The gut mucosa is a complex organ that can be divided into several compartments: the epithelial layer, located in the luminal part; and the lamina propria layer, lying underneath the epithelium. Each compartment is composed of a mixture of cell types, which may have specialized and local functions, and that are distributed differentially across the gastrointestinal tract^10,11^. On the luminal side, the gut mucosa is composed of an a-cellular mucus layer, separating gut bacteria from the epithelium. This layer functions as a second barrier between the outside world and the inner gut compartment. Directly underneath is the epithelial layer, in which immune cells infiltrate and perform specific functions for antimicrobial defense and antigen signaling. One of these immune cell populations that only resides here is the intraepithelial lymphocyte population (IEL), which is functionally different from the lymphocytes in the lamina propria (LPL), the underlying layer. The latter layer also contains other immune cells, vasculature, nerves and mesenchymal cells^12–14^.

To effectively profile single cells of such a complex and solid organ, the tissue needs to be dissociated, which is an essential first step in every scRNA-seq protocol. However, several major hurdles are hampering the upscaling of single-cell gut atlases. First, the duration of the dissociation and the procedure itself can introduce unwanted artifacts in the downstream gene expression read-out^15,16^. Each step in the dissociation procedure may influence gene expression, hence processing should be limited to what is strictly necessary. Second, the dissociation procedure should be sufficiently thorough, separating all cells without damaging more fragile cell types. This is especially true if the researcher wants to study an accurate reflection of the original tissue, such as for the use of cell atlases. This balance between obtaining a representative group of viable, single cells, and minimizing artifacts requires fine-tuning.

To obtain this accurate reflection of the original tissue, the current dissociation procedures of gut biopsies can be improved in two ways. Firstly, by avoiding differences between cells from the same tissue as induced through multi-step digestion: ideally one would process the whole tissue using a one-step dissociation procedure. The current gold standard for isolating single cells from the gut, however, is a two-step dissociation procedure: the epithelial layer is dissociated first, using EDTA, followed by the lamina propria layer, using a 37 °C collagenase treatment (henceforth referred to as the two-step collagenase protocol)^17–19^. Secondly, current scRNA-seq protocols on gut tissues have previously been limited to fresh gut material, either collected through biopsy during colonoscopy or as resection material from a surgical procedure. As a result, only a very limited number of samples can be processed at a time, which may introduce batch effects between samples without the possibility to correct for this. To answer these concerns, a recently developed cryopreservation procedure of gut mucosal tissue now enables conducting multi-gut-sample experiments with living cells^20,21^.

To overcome these limitations, we have developed a novel gut dissociation protocol (one-step collagenase dissociation) suitable for scRNA-seq protocols that treats all cells the same way, aiming to prevent batch effects between different cell types that are otherwise isolated sequentially. We first show that cryopreservation is not affecting sequencing quality parameters nor cell viability of the one-step collagenase dissociated cells, but presumably impacts the cell type composition and gene expression levels. We then compare sequencing parameters, cell viability, cell yield and gene expression of scRNA-seq and FACS using this one-step collagenase dissociation on cryopreserved biopsies with the gold standard gut tissue dissociation method (two-step collagenase protocol) and another alternative using a three-step digestion with a protease isolated from *Bacillus licheniformis* that is active at 4 °C (three-step protease) [adapted from^22^and^23^]. The latter protocol aims to minimize gene expression artifacts generally associated with dissociation, by keeping cells metabolically inactive at 4 °C. Moreover, it was designed to prevent death of anoikis-prone intestinal epithelial cells in the healthy intestine for scRNA-seq studies. In the first step of this procedure, biopsies are incubated on ice with a low concentration of EDTA to release loosely bound immune cells (fraction 1), whereafter the epithelial layer, containing infiltrating immune cells, is dissociated using cold-active protease (fraction 2) and both fractions are treated briefly with collagenase IV to ensure complete digestion. By comparing effects on cell viability, cell type composition and single-cell gene expression levels, we determined the best-use scenarios and the limitations for each protocol. We then summarized this in a protocol decision tool that can help scientists decide which protocol best suits their biological questions.

## Results

### Experimental design

**Figure 1:**
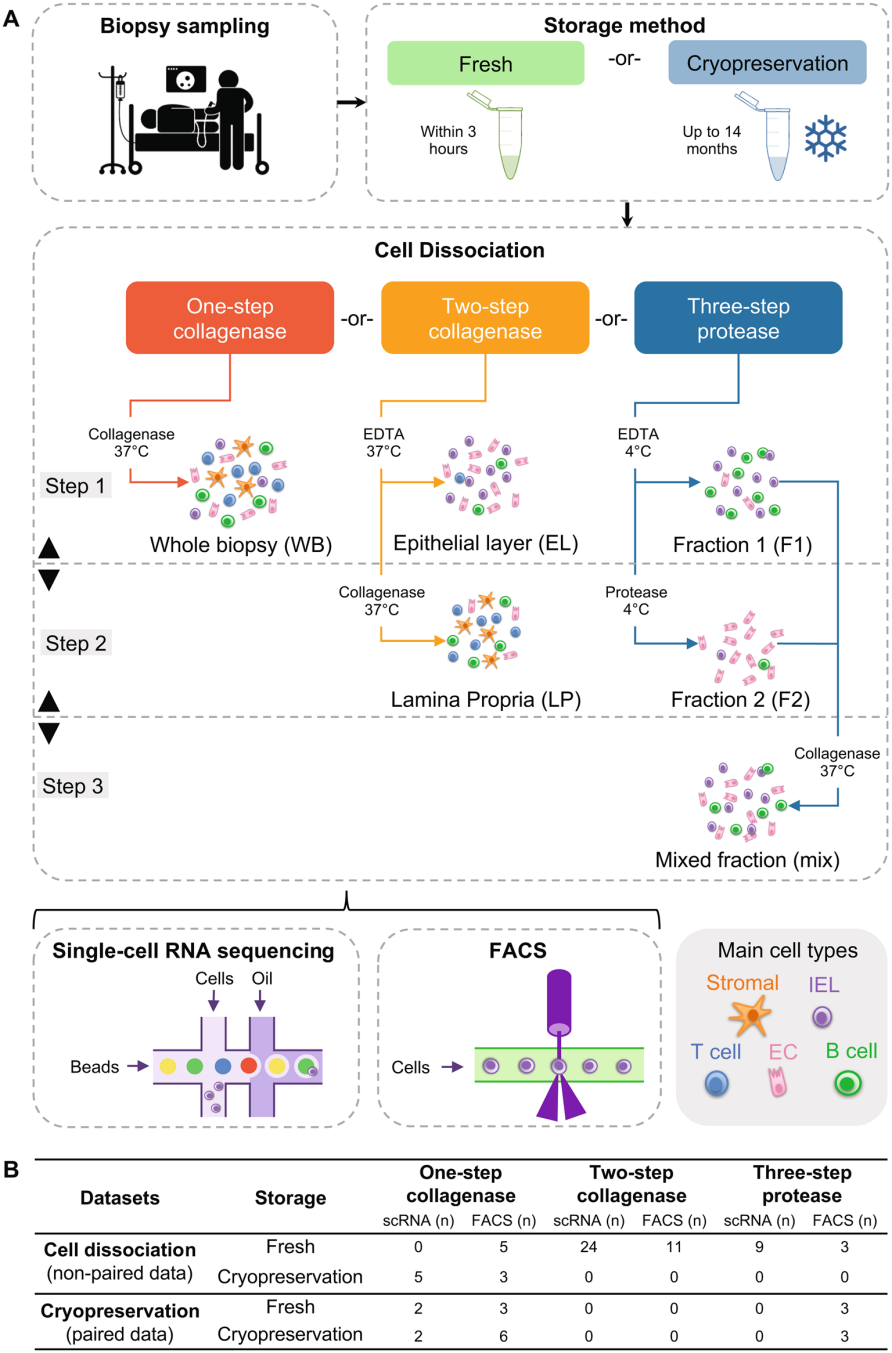
Study overview and datasets. (**A**) Schematic representation of the study set-up. Biopsies were sampled, either used fresh or cryopreserved, and dissociated using the one-step collagenase protocol with collagenase at 37 °C; the two-step collagenase with EDTA in step 1 and collagenase at 37 °C in step 2; or the three-step protease protocol, starting with 4 °C EDTA incubation in step 1, protease digestion in step 2, and a short collagenase digestion at room temperature in step 3. (**B**) Two datasets were generated to investigate: 1. the effect of cryopreservation and 2. the effect of different dissociation protocols on cell-type viability, composition, transcriptome, and functionality. IEL, intraepithelial cell; EC, epithelial cell. Colonoscopy pictogram was drawn by Gan Khoon Lay, https://thenounproject.com/term/colonoscopy/1250282/.

To assess the effect of different dissociation protocols in different cell types simultaneously and with high resolution, a side-by-side comparison was performed using both flow cytometry (FACS) and scRNA-seq data (Fig. 1A). Two separate datasets were used to either determine the effect of the cryopreservation or the cell dissociation protocol (Fig. 1B). For scRNA-seq, the effect of cryopreservation was compared on two paired samples using the one-step collagenase protocol, whereas the effect of dissociation on cell viability, cell type composition and gene expression was compared on (a larger set of) samples from different individuals. For FACS, cryopreservation was assessed on the one-step collagenase and the fraction 2 of the three-step protease protocols, whereas the impact of cell dissociation was evaluated on a larger dataset. Each protocol resulted in different compartments (Fig. 1A):

- The one-step collagenase protocol-derived data was analyzed for cell type composition as a whole, from here referred to as whole biopsy (WB) compartment.
- The two step collagenase was analyzed in two parts: the epithelial layer (EL) and lamina propria (LP) fractions, respectively.
- The three step protease protocol was analyzed using scRNA-seq as both a 50/50 mix of fraction 1 (F1) and 2 (F2), and as fraction 2 only, and using FACS as fraction 1 and 2 separately.

### Impact of storage conditions on cell viability, cell type composition and scRNA-seq library quality

**Figure 2:**
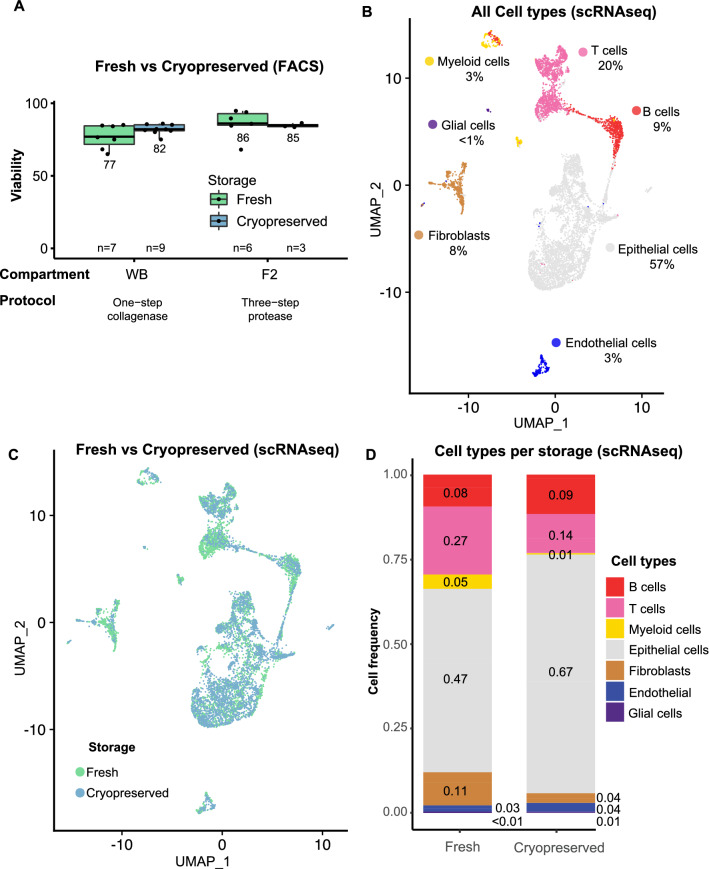
Impact of cryopreservation on cells dissociated using the one-step collagenase protocol in FACS and scRNA-seq. (**A**) Boxplots showing FACS cell viability (%) in dissociated cells from fresh versus cryopreserved samples for whole biopsy (one-step collagenase protocol) and fraction 2 (three-step protease protocol) (p-value > 0.05; non-paired t-test). Sample size is depicted in the lower part of the plot. UMAPs of scRNA-seq data obtained from two-paired fresh and cryopreserved samples dissociated with one-step collagenase colored by (**B**) cell types and (**C**) storage condition. (**D**) Bar plot of relative cell type frequencies recovered from the scRNA-seq data colored by cell-type.

To assess the impact of cryopreservation on the samples, we compared the cell viabilities obtained after dissociation using the one-step collagenase and the three-step protease protocols by a cell viability dye for flow cytometry. We found that the cell viability of the samples from both protocols showed similar viabilities (median 81.5%, SD 12.8 Fig. 2A, Suppl. Table 1). Although the mean of the cryopreserved samples from the one-step collagenase was higher than the fresh samples, differences were non-significant (p > 0.05, non-paired t-test).

To investigate the effect of cryopreservation on scRNA-seq after the one-step collagenase protocol, we used paired fresh and cryopreserved samples from two individuals. Firstly, basic parameters for sequencing quality control showed that RNA integrity, as measured by the quality of the sequenced reads, the mappability of the reads to the genome and the cell QC parameters, was not affected by the cryopreservation procedure (Suppl. Table 2). Next, we clustered the cells based on their transcriptome and determined the cell type identity as described in Methods (Fig. 2B). The fraction of mitochondrial genes was assessed per cell category (epithelium, stromal, immune), since a cell-type- dependent difference in mitochondrial gene counts exists. Stromal and epithelial cells show a trend toward higher mitochondrial gene expression after cryopreservation, whereas immune cells appear lower in mitochondrial gene counts (Suppl. Figure 1A–D). When comparing the fraction of mitochondrial genes at a higher cell type resolution, we observe that T cells show a clear bimodal distribution in fresh samples, which could indicate the recovery of low quality cells (Suppl. Figure 1E).

By using a UMAP as visualization, we observed that fresh and cryopreserved cells are relatively well distributed among the cell types (Fig. 2C). To reveal how the cellular composition is affected by the storage mode, we used both scRNA-seq data (Fig. 2D) and flow cytometry (Suppl. Figure 2). In the scRNA-seq data, we noted suggestive evidence of a decrease in the proportions of myeloid cells (5–1%) and fibroblasts (11–4%), and an increment in epithelial cells (47–67%) (Fig. 2D). Similarly, after cryopreservation we observed a significant decrease in the cell proportions of myeloid cells (~ 5–2%) as well as B cells (~ 13–6%, not observed in scRNA-seq data), whereas the proportion of epithelial cells increased (~ 38–55%) (Suppl. Figure 2).

In summary, we observed for both protocols, one-step collagenase and three-step protease, that cryopreservation (verified up to 14 months) did not significantly impact the cell viability. We found suggestive evidence that overall cell type composition was minorly affected by cryopreservation, with the exception of myeloid cells, fibroblasts and B cells, which were presumably reduced after cryopreservation (Fig. 2D, Suppl. Figure 2), consistent with previous reports^20^. In conclusion, cryopreservation of gut biopsies allows for long-term storage and higher-throughput experimental setups. Therefore, combined with the absence of any major detrimental effects on cell viability, cell type composition or gene expression levels, this is the sample processing method of choice.

### Effect of dissociation protocols on cell viability and scRNA-seq integrity

**Figure 3:**
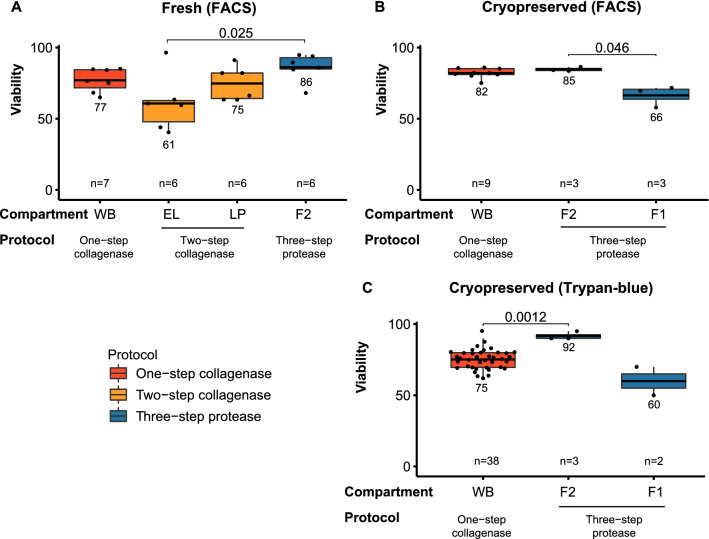
Effect of dissociation protocols on cell viability assessed by FACS and hemocytometry. Boxplots comparing cell viability (%) in (**A**) fresh samples measured by FACS; and cryopreserved samples measured by (**B**) FACS and (**C**) trypan-blue exclusion. Compartments and dissociation protocols are indicated on the x-axis. The average cell viability was compared between compartments (non-paired t-test). Only significant comparisons (p-value < 0.05) are shown as a bar, and the respective p-value, above the compared compartment. Sample size is depicted in the lower part of each plot. WB, whole biopsy; EL, epithelial layer; LP, lamina propria; F1, fraction 1; F2, fraction 2.

Next, we explored the impact of the cell dissociation protocol on the viability of cells. In fresh samples, the highest cell viability was achieved in fraction 2 of the three-step protease protocol, which is mainly composed of epithelial and immune cells, but it only reached a significant difference when compared to the EL fraction of the two-step collagenase protocol (Fig. 3A, p < 0.05). The second highest viability was achieved by the one-step collagenase protocol (WB, whole biopsy) and the LP (lamina propria, mainly immune cells) obtained by the two-step collagenase protocol (Fig. 3A, Suppl. Table 1). We observed that the EDTA-only treated epithelial cells in the two-step collagenase protocol showed the lowest viability (Fig. 3A, Suppl. Table 1). However, these comparisons were non-significant (p < 0.05).

In cryopreserved samples, we compared the WB from the one-step collagenase protocol and both fractions from the three-step protease protocol. Our results suggest a compromised cell viability (~ 60–66%) on fraction 1 of the three-step protease protocol (p < 0.05 by FACS, Fig. 3B; inconclusive by trypan-blue exclusion test, Fig. 3C).

Lastly, we investigated the fraction of mitochondrial genes out of total gene expression in all protocols. We found that the one-step collagenase protocol has an elevated fraction of mitochondrial gene expression as compared to the other protocols, being an indicator for elevated levels of cell death^24^ (Suppl. Figure 3A–H).

### Effect of dissociation protocols on cell type composition

**Figure 4:**
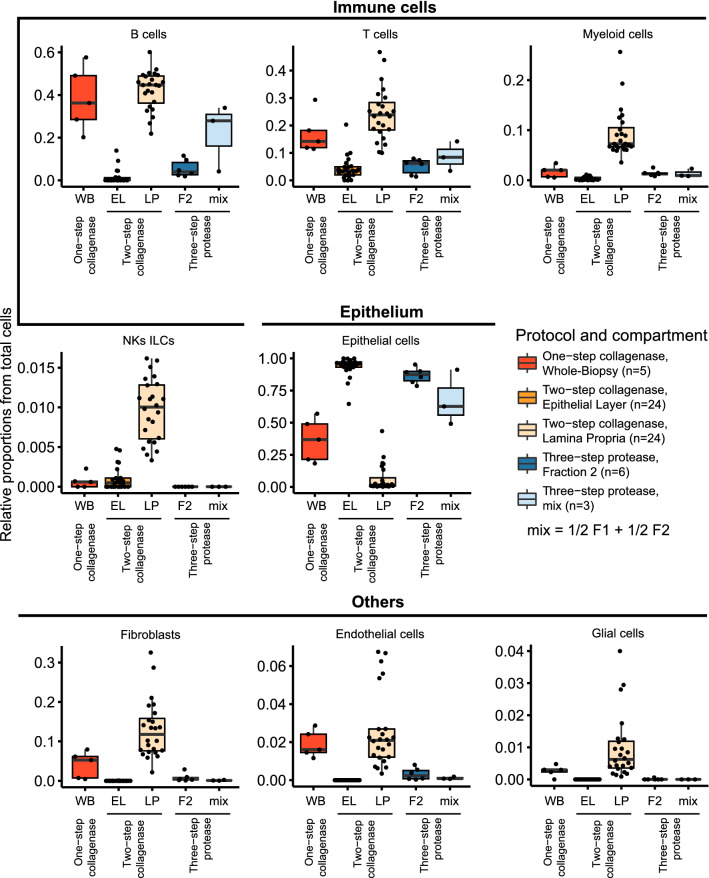
Differences in cell type proportions recovered in each protocol using scRNA-seq. Boxplots of cell type proportions characterized by scRNA-seq. The resulting cell type composition of samples was tested across the protocols and compartments (Wilcoxon rank sum test with Holm’s correction for multiple testing, Suppl. Table 6). Compartments and dissociation protocols are indicated in the x-axis. Sample size is depicted in the legend of the plot. WB, whole biopsy; EL, epithelial layer; LP, lamina propria; F1, fraction 1; F2, fraction 2.

The one-step collagenase dissociation resulted in a balanced recovery of epithelial and immune cells that also included other cell types such as fibroblasts, endothelial cells and glial cells (Fig. 4, Suppl. Figure 4). In contrast, both multi-step protocols supported isolation of specific cell types in either of the two isolated compartments (Fig. 4, Suppl. Figure 4). The three-step protease protocol supports viability of epithelial cells, which make up a large fraction of intestinal cell types, and so rarer cell types such as NKs ILCs (a mix of natural killer and innate lymphoid cells), endothelial and fibroblast cells appear underrepresented by comparison in scRNA-seq data. For example, by FACS, NKs ILCs are best captured in fraction 1 of the three-step protease protocol (Suppl. Figure 4; ~ 5% vs. < 1% for collagenase protocols) but appear missing in the scRNA-seq data set (Fig. 4).

As specific research questions may require the isolation of specific cell types, we assessed which protocol best supported the isolation of each of the major cell types with the highest cell viability. The epithelial layer of the two-step collagenase protocol and fraction 2 of the protease protocol support isolation of epithelial cells better than the one-step collagenase protocol (p-value < 0.05, Fig. 4, confirmed by flow cytometry, Suppl. Figure 4). On the other hand, fraction 1 of the three-step protease protocol provides the most purified immune population (Fig. 4), albeit with suggestive compromised cell viability when using cryopreserved biopsies (Fig. 3B–C), and reduction of layer-specific immune cells such as the CD8 + LPL T cells (Suppl. Figure 6). However, for the best separation of layer-specific immune cells, the two-step collagenase protocol is the protocol of choice. Despite this, variable separation of the compartments remains an issue with all multi-step dissociation protocols and is a potential source of bias. This variability can be caused by multiple factors, such as incubation time, centrifugation speed, concentration of enzymes and chemicals, temperature, surface area of tissue, and intrinsic donor variability^25^. Therefore, applying combinatory scRNA-seq and surface protein sequencing (CITE-seq)^26^ to one-step collagenase dissociated biopsies can be a good alternative that enables studying layer-specific immune populations, such as the CD103 + IEL cells, without introducing layer-specific batch effects. Moreover, other strategies for enrichment, such as bead enrichment and FACS, may provide this cell type enrichment without being limited to a specific dissociation protocol, although requiring extra laboratory steps.

Summarizing, the one-step collagenase protocol captures the gut composition with a balanced cell viability, whilst the multi-step collagenase and protease protocols are preferable when focusing on specific compartments or cell types.

### Effect of dissociation protocols on gene expression

Different cell types can respond differently to chemical treatments, thereby harboring potential batch effects. To determine whether the different dissociation protocols induce such batch effects, we performed differential gene expression (DE) and pathway analysis on paired fresh versus cryopreserved scRNA-seq samples dissociated with the one-step collagenase protocol (Suppl. Table 2 and 3a + b).

Previous studies indicate that 10–50% of the transcriptome is affected by dissociation^27^. Therefore, similar dissociation treatments for all cell types, as in the one-step collagenase protocol, will reduce the DE between cells that is induced by differential dissociation treatments. To quantify the effect of differential treatment, we performed DE analysis on the epithelial cells that were captured in the EL vs LP fraction of the Smillie et al. dataset^17^. Studying these epithelial cells present in both fractions of the two-step protocol allows for quantification of the effect of the differential treatment between the EL (EDTA only) and LP (EDTA, followed by collagenase) fraction. This DE analysis revealed 222 DE genes, of which 34 were previously described to be collagenase-induced in collagenase-dissociated muscle stem cells from mice^15^. Notably, each of those 34 collagenase-induced genes were found upregulated in the LP fraction (i.e. the fraction treated with collagenase). Together, this analysis reveals a clear batch effect between the EL and LP fraction that was induced by the differential treatment, and indicates an enrichment for previously described collagenase-induced genes (34/222 collagenase-induced genes in the DE gene list as opposed to 104/3947 that were in the non-DE genes, Fisher Exact p < 0.00001). It is this batch effect of the differential treatment that is circumvented using the one-step collagenase protocol.

We compared collagenase-treated epithelial cells of the one-step protocol on the one hand with the EDTA-treated cells from the two-step collagenase on the other hand with the EDTA + protease + collagenase-treated cells from the protease protocol. We found that the epithelial cells of the one-step collagenase protocol show upregulation of 95 (10% of total DE genes) and 60 (7% of total DE genes) known collagenase-induced genes^16^ (Suppl. Table 3a), respectively, including genes such as FOS and JUN (Suppl. Table 3a + b, Suppl. Figure 5). From these 95 and 60 DE genes, only a total of 13 collagenase-induced genes is overlapping, indicating that collagenase may not be the only factor contributing to gene expression differences here.

The immune cells (myeloid cells, B and T lymphocytes) revealed mostly similar numbers of DE genes between the one-step collagenase protocol and the multi-step protocols, except for the B cells. In these cells we observed a large discrepancy in the number of upregulated versus downregulated genes in the one-step collagenase versus multi-step protocols; 976 genes are downregulated in the two-step versus the one-step collagenase protocol, whereas 90 genes are upregulated (Suppl. table 3a).

Remarkably, myeloid and B cells show the highest proportion of collagenase-induced genes upregulated in the one-step collagenase as compared to the three-step protease protocol (27% and 25%, respectively) (Suppl. Table 3a)^16^. This indicates that these cell types may be most sensitive to collagenase digestion time, possibly influencing the cell subtype composition of the cell type, for example mostly plasma cells versus mostly B cells (Suppl. Figure 4). There is a trend towards higher proportion of B cells, and lower proportion of plasma cells in protease as compared to one-step collagenase digested cells in FACS, supporting this theory (Suppl. Figure 4). Furthermore, it implies that many genes are affected by other factors than the enzyme used for digestion such as the digestion duration and temperature differences.

Overall, the lamina propria layer from the two-step collagenase protocol, which is treated with collagenase I + II-containing liberase, contains most myeloid cells (Fig. 4, Suppl. Table 6). However, we expected similar proportions of myeloid cells in collagenase IV-treated one-step collagenase dissociated biopsies, as the enzymatic digestion and its duration are otherwise comparable. It has been shown that dissociation enzymes influence cell subtype dissociation efficacy^28^. The lower myeloid cell number in the one-step collagenase protocol as compared to the two-step collagenase protocol may be a result of cryopreservation of the one-step collagenase dissociated samples^20^ (Fig. 4, Suppl. Table 6).

Amongst the pathways upregulated in T cells after one-step collagenase digestion versus three-step protease digestion, ‘signaling by interleukins’ and ‘cellular responses to external stimuli’ come up, suggesting that the warm collagenase protocol may indirectly activate T cells, or that cold protease digestion may prevent immune cell activation during isolation (Suppl. table 3b).

Fibroblasts showed the most DE genes in both comparisons against the one-step protocol: 2,184 genes were DE with the two-step collagenase protocol and 1,833 with the three-step protease protocol. Interestingly however, amongst these DE genes, there was only a small fraction of collagenase-induced genes (8% and 10%, respectively) (Suppl. Table 3a). This implies that fibroblasts are not necessarily sensitive to collagenase treatment, but probably other chemicals used for dissociation have a larger impact (i.e. EDTA).

### One-step collagenase dissociated cells from cryopreserved biopsies provide a source of functional cells

To demonstrate the feasibility of collecting viable and functional single cells after the one-step collagenase digestion on cryopreserved biopsies, we demonstrated that both gut organoids (Suppl. Figure 8A) and IELs (Suppl. Figure 8B–C) can be successfully propagated from the remaining cells after scRNA-seq. For IEL propagation, the remnant cells were FACS sorted and grown for 10 days. In this way, IELs could be expanded up to 30 times from the starting material while keeping their phenotype (Suppl. Figure 6). These findings underline the possibility to study both functionality and gene expression of cells from the same samples using cryopreserved biopsies. Altogether, these results open the possibility to design single-cell omics experiments followed by experimental validation from the same biological material, thereby reducing the batch effects and facilitating the retrieval of multiple layers of information from samples with the same genetic and environmental context.

## Discussion

**Figure 5:**
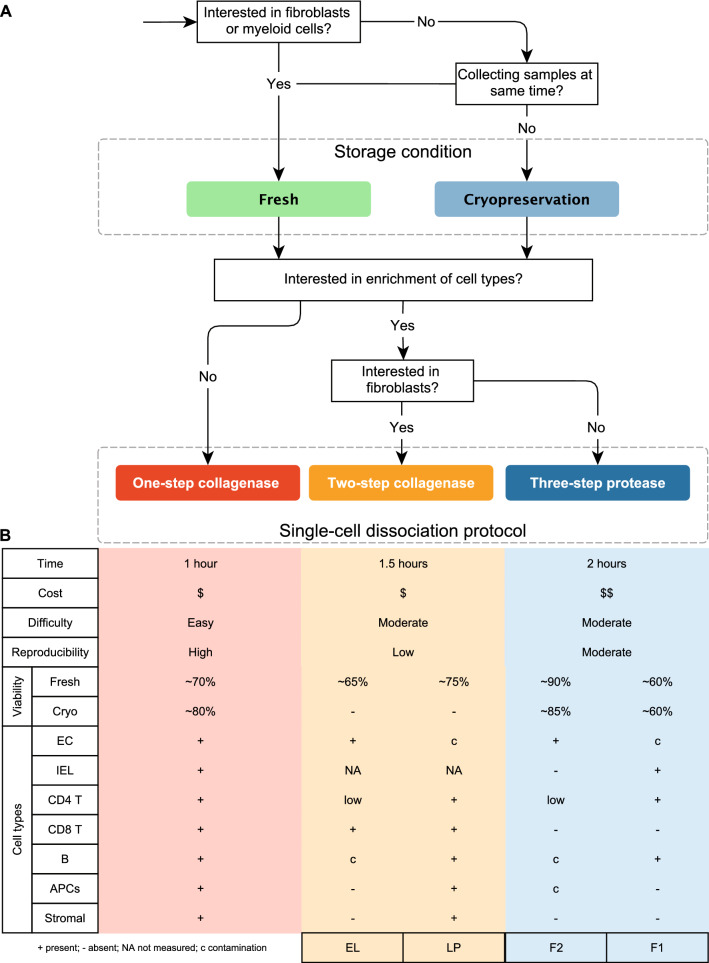
Decision tree for single-cell dissociation of gut tissue for scRNA-seq. (**A**) Flow-chart designed to assist researchers in choosing a dissociation protocol for gut tissue based on their specific needs. (**B**) Overview of the main differences between the protocols. IEL, intraepithelial cell; EC, epithelial cell; EL, epithelial layer; LP, lamina propria; F1, fraction 1; F2, fraction 2.

In this study, we present a one-step collagenase gut biopsy digestion protocol as a well-grounded alternative for the current gold standard two-step collagenase dissociation^17,19,29^ and three-step protease dissociation [adapted from^22^ and^23^]. The main advantages of this one-step protocol are the reduced time, cost and proceedings, the prevention of batch effects associated with the sequential dissociation of different compartments in the multi-step protocols, combined with a high level of reproducibility and experimental flexibility (Fig. 5). Furthermore, we show that this protocol generates viable single cells, also after cryopreservation, that are suitable for functional studies using organoids or downstream analysis using scRNA-seq or FACS.

Cryopreserving biopsies allows for more efficient processing, and therefore, a reduced number of experimental batches. As fresh biopsy material is rarely collected from multiple donors at the same time, fresh material is usually processed one sample at a time. In contrast, cryopreservation tremendously increases the number of samples that can be processed concurrently, as well as intermixing samples from different timepoints and patients; with the one-step collagenase protocol one can process 8 samples per person within 1 h. Furthermore, cryopreservation enables waiting for pathology reports of the (adjacent) tissue, as well as investigating the inflammation or malignancy status of the samples before sample processing. This can be highly advantageous when studying the same patients longitudinally and while trying to capture samples around a specific associated clinical event. This targeted sample selection may limit processing unsuitable samples, thus further limiting costs. Previously, cryopreservation has shown to result in viable cells suitable for scRNA-seq in diverse tissues, such as kidney^30^, pancreas^31^ and synovial^32^ tissue. To the best of our knowledge, for gut mucosa, such a study has not been conducted so far. Moreover, Konnikova et al. showed that cryopreservation of gut mucosa preserves high cell viability and functionality, implicating the suitability of cryopreserved gut mucosa tissue for scRNA-seq^20^. In the limited number of two paired samples, we see that beyond the good cell viability and functionality, cryopreservation has only minor effects on cell type composition and gene expression and is suitable for use in scRNA-seq analysis.

Despite the clear advantages of cryopreserving samples and using the one-step over the multi-step protocols, there are specific cases in which other experimental set-ups are more optimal. For example, a multi-step dissociation protocol may be preferable for optimizing yield of layer-specific cell types, or when surface proteins to mark such cell types only present in a certain layer are not available. Additionally, while cryopreservation brings many advantages, the loss of myeloid cells dictates the use of fresh material when this is the main cell type of interest.

In summary, when deciding for a dissociation protocol, one needs to weigh many factors, including cell composition, cell viability and batch effects. Depending on the research questions asked, the importance of these factors might be shifted. Therefore, the best dissociation approach for all possible situations does not exist. Based on the comparisons in this study, we have developed a protocol decision tool, which may guide scientists through several questions in a decision tree to help decide for the optimal dissociation strategy for their experimental setup and questions (Fig. 5).

By propagating epithelial and immune cells using cryopreserved and dissociated cells, we showed the versatility of the one-step protocol for use in other experiments than scRNA-seq. We believe that multiple layers of data can be obtained from the same biological material, allowing the application of both functional validations and high-throughput techniques. For example, based on our results showing the presence of surface markers after dissociating cells using either protease or collagenase, cells can be used in a method studying both surface marker and single cell transcription at the same time, such as CITE-seq. Another method that can be applied is CyTOF, which enables the exploration of around 40 cell surface markers at same time in millions of cells, enabling surface marker identification of (rare) cell types^33^. As demonstrated, if handled properly, cells can be isolated and propagated, thereby allowing further functional analysis. Altogether, the here-presented one-step collagenase dissociation protocol using cryopreserved biopsies may have positive repercussions in personalized medicine. Firstly, specific tissues in a disease context can be deeply characterized to identify pathogenic cell types. Secondly, these pathogenic cells would allow the discovery of new drug targets for therapies and may even be used to evaluate the efficacy of such drugs^34^.

One of the limitations in all cell dissociation protocols is that the spatial configuration of cells is lost. As aforementioned, gut is a complex tissue where location plays a major role in determining how specific cells behave. Currently, there are novel developments such as imaging mass cytometry^35^ and spatial transcriptomics^36,37^, which can help study cell localization in the tissue. In conjunction with deep single-cell multi omics techniques, characterizing the interactome of gut in complex diseases may open the doors to new advances in medicine.

In addition to the single-cell dissociation methods compared in this manuscript, single-nuclei isolation methods could become a suitable alternative to avoid batch effects associated with the method of dissociation or in case only frozen tissue is available^38,39^. When comparing the nuclear with the cytoplasmic transcriptome of individual cells, correlations are high but the number of UMIs captured in the nuclei will always remain a fraction of what can be captured in a single-cell^40,41^. On top of that, currently, no protocol exists that can efficiently isolate nuclei for scRNA-seq from the full variety of cell types residing within the highly heterogeneous tissue of the gut. Nevertheless, when such protocols become available they could become an interesting alternative for cryopreserved single-cell dissociation methods.

We present a novel gut mucosal tissue dissociation protocol which will enable future scientists to conduct in plannable, large scale (multi-center) research with limited batch effects. There have been few studies comparing dissociation protocols for gut mucosal tissue dissociation, let alone for subsequent scRNA-seq, which underlines the importance of the findings presented here. Considering the composition of the gut mucosa, we believe the methodology described in this paper could be a good starting point for the dissociation and cryopreservation of other mucosal tissues, such as lung or oral mucosa, for the purpose of single-cell sequencing studies. In addition, the results may inspire researchers from other fields to question factors influencing their scRNA-seq data.

## Methods

### Biopsy collection, cryopreservation and thawing

Biopsies were collected in RPMI1640 supplemented with GlutaMax (Gibco) and 5% heat-inactivated FCS. After collection, they were kept on ice for a maximum of 30 min. Biopsies were either used directly for dissociation (fresh) or cryopreserved in cold freezing medium (90% heat-inactivated FCS with 10% DMSO). Cryopreservation of biopsies and thawing procedures were performed as described before^20^. In short, biopsies were quickly (< 60 s) thawn in a 37 °C water bath and washed twice with cold thawing solution (PBS without MgCl2 and CaCl2, supplemented with 5% heat-inactivated FCS). We generated two datasets: a ‘cryopreservation dataset’, using paired fresh-cryopreserved samples from 2 individuals dissociated with the one-step collagenase protocol only, and a non-paired ‘cell dissociation’ dataset, using gut biopsies from different individuals isolated using all dissociation protocols described here-below. For the ‘cell dissociation’ dataset we used two-step collagenase-digested cells from the Smillie et al. dataset^17^.

### Biopsy dissociation

Biopsies were dissociated following one of these three protocols:

1. Two-step collagenase protocol, using fresh biopsies and collagenase I + II-containing liberase and splitting IEL and LPL layer, as described previously^17^
2. Three-step protease protocol, using fresh biopsies and cold protease as described previously, splitting F1 and F2 [adapted from^22^ and^23^]
3. One-step collagenase protocol, using cryopreserved biopsies and collagenase IV to isolate biopsies as a whole (WB) in one digestion step.

In short, the two-step collagenase protocol incubates fresh biopsies for 15 min in RPMI1640 with EDTA on a rotisserie rack at 37 °C, after which thorough shaking sheds the epithelial layers off the lamina propria. Then, the split layers are digested separately while rotating at 37 °C for 30 min: the epithelial layer is digested with TrypLE Express Enzyme (Gibco) and the lamina propria layer with a mixed collagenase preparation (Liberase TM).

The three-step protease protocol first cuts biopsies into small pieces. Subsequently, pipette-mixing in cold (4 °C) HBSS supplemented with EDTA separates fraction 1, which is mainly immune cells. These are removed in the supernatant, and the epithelial and lamina propria cells remain in tissue clumps. The epithelial layer and lamina propria are then dissociated using a 30 min incubation in HBSS with cold (4 °C) protease and EDTA, and subsequently with a 10 min collagenase digestion at RT to dissociate the final tissue clumps (fraction 2). All cells are then incubated with ACK lysis buffer to remove sub-viable cells before two final washes.

The one-step collagenase protocol incubates biopsies for 25 min at 37 °C in a shaking incubator in digestion medium (RPMI1640 supplemented with GlutaMax (Gibco), 200 iU/mL collagenase IV C1889 (ThermoFisher), 10 iU/mL DNAse II D8764 (ThermoFisher), 35 iU/mL SUPERaseIn AM2694 (Invitrogen), 2% heat-inactivated FCS). Optimally, 2–4 pinch-biopsies are dissociated per 2.5 ml digestion medium. After 10 min of incubation in digestion medium, the biopsies are resuspended with a p1000 pipette tip. If a biopsy does not digest well (rectum is generally tougher than ileum, fibrotic tissue can be more difficult), one may cut it with (cleaned, sterilized) scissors and/or cut the pipette tip to enable a larger pipetting lumen. After an additional 15 min of incubation, the biopsies are pipette-strained again using a p1000 tip, followed by a p200 tip. Next, the digestion is stopped by adding 5 mM EDTA (Sigma) for 2 min on ice. Then, > 10 ml of RT PBS−/− is added to minimize residual effects of the collagenase-EDTA reagents, after which cells are centrifuged for 5 min at 300 g at RT. The pellet is resuspended in 100ul TrypLE Express Enzyme and incubated for 1.5 min in a 37 °C water bath. The enzymatic reaction is stopped by adding cold RPMI1640 with 2% heat-inactivated FCS and the cells are centrifuged for 5 min at 300 g at 4 °C. Next, the pellet is resuspended in cold wash buffer I (PBS -/- supplemented with 0.4% BSA and 10 iU/mL DNase II) and filtered through a 70 um cell strainer. To maximize cell collection, the filter is washed once more with cold wash buffer II (PBS -/- supplemented with 0.4% BSA) and the cells are collected after centrifugation for 5 min at 300 g at 4 °C.

### Cell hashing

To allow multiplexing, which may prevent effects induced by differences between sequence batches, dissociated cells from separate samples are labelled with barcoded antibodies (‘cell hashing’) using TotalSeq-A oligo-conjugated hashtag antibodies (Biologend) following the manufacturer’s protocol [Stoeckius et al. 2018]. In short, 400.000 cells are incubated for 30 min on a rocking plate on ice with 0.5 μg hashtag antibodies and 100 ul staining buffer. After this, cells are washed twice with PBS^−/−^ supplemented with 0.4% BSA to remove excess hashtags. By doing so, the cryopreserved samples, dissociated with the one-step collagenase protocol, are hashed separately and pooled per 4 samples per 10 × lane pool. Fresh samples are collected, dissociated and loaded separately in a single 10 × lane.

#### Cell viability before loading on 10X Genomics Chromium Controller

Cell viability was determined pre-loading on a 10X Genomics Chromium Controller by staining cells with trypan blue staining reagent (Sigma, T6146) 1:1, and counted using a hemocytometer. % viability was calculated as [number of stained cells] divided by [number of all cells] *100%.

### Single-cell library prep and sequencing

Hashtag and cDNA libraries were generated using Biolegend’s instructions in combination with the protocol accompanying the 10X Genomics library kit (v2 and v3 for the one-step collagenase protocol, and v3 and v3.1 for the three-step protease protocol, respectively). These dissociation procedures were compared with previously published data that was collected using the two-step collagenase protocol^17^, generated using 10X Genomics library prep v1 and v2. Libraries containing samples of the one-step collagenase protocol were in part sequenced at BGI (Hong Kong) on an Illumina NovaSeq6000 using a 150 bp PE kit and on a BGISEQ500 using a 100 bp PE kit using custom programs (28-8-150 bp and 100-8-150 bp, respectively). Libraries were sequenced to an average of 110,442 (SD 42,975) reads per cell for the cDNA libraries and 3,073 (SD 1,768) reads per cell for the hashtag libraries. Libraries containing samples of the three-step protease protocol were sequenced at Sanger Institute (Hinxton, UK), partly on a HiSeq 4000 75 PE kit and partly on a NovaSeq S4 100 PE XP machine. Protease libraries were sequenced to an average of 103,181 (SD 34,489) reads per cell for the cDNA library.

#### Basic processing and QC filtering of scRNA-seq and hashtag data

The CellRanger v3.0.2 mkfastq and count pipelines were used to make FASTQ files, align the sequencing reads to the hg19 reference genome for one-step collagenase samples and to the hg38 reference genome for protease samples, filtering of cell and UMI (unique molecular identifier) barcodes, and counting gene expression per cell. Downstream data analysis was performed using Seurat v3.1.4. The count matrix was filtered for cells containing < 200 genes and > 60% mitochondrial reads. Hashtag data was normalized using centered-log ratio transformation and HTODemux was used to assign a hashtag ID to each cell. Only single, hashtag-IDed cells were selected for further analysis.

### Cell type classification

Clusters were defined using various resolutions, aiming to fit the cell types present in the data. Cell type classification was performed using a two-step approach. First, the scPred package^42^ was used to train an SVM prediction model on a subset of 750 cells per cell type of the source data from the two-step collagenase protocol^17^, which was filtered and prepared using the same QC steps as described above. This model was used to predict coarse cell types for samples generated with each of the other two protocols. Second, datasets of all three protocols were integrated using SCTransform integration^43^, while regressing out the proportion of mitochondrial and ribosomal genes. The first 30 principle components of this integrated dataset were used for cell clustering using Seurat’s FindCluster function and a UMAP was used to visualize this. Cell type specific markers for the resulting cell types are depicted in Suppl. Figure 9.

#### Data quality assessment cryopreservation and dissociation datasets

The cryopreservation dataset was used to assess the effect of fresh use versus cryopreservation on basic sequencing quality parameters. First, we made an overview of sequencing parameters from 1 of the fresh samples from the cryopreservation dataset versus 1 paired cryopreserved sample of the cryopreservation dataset and 5 equally processed cryopreserved samples from our lab.

Next, for the cryopreservation and the dissociation datasets, we assessed the quality of the data by querying the relation between the number of genes expressed and the number of UMIs present in each dataset. Second, we visualized mitochondrial gene expression versus number of UMIs present in the dataset.

#### Differential expression analysis

DE analysis was performed using MAST through Seurat’s FindAllMarkers function^44^. For this, library scaled (10,000 transcripts per cell), log-2 transformed RNA expression data was used as input. Only genes present in at least 10% of cells belonging to either one of the investigated clusters and with a log fold change of at least 0.25 were tested. Only comparisons of > 100 cells per group were included in the analyses, and only genes present in all of the compared groups were included in the subsequent pathway analyses.

#### Assessment of collagenase-induced batch effect

To assess the influence of collagenase treatment on gene expression, epithelial cells from the Smillie et al. dataset^17^ deriving from the first and the second step of the protocol, respectively, were compared. First, cells were subset from the main dataset. Since size differences between the two steps might skew the analysis, cells were downsampled to a matching number of 2617 cells per dissociation step. Differential expression analysis was performed as described below, and resulting genes were scanned for overlap with a set of 140 collagenase-induced genes^15^. Fisher’s exact test was performed to assess whether the differentially expressed genes were enriched for collagenase-induced genes.

#### Pathway analysis

Upregulated pathways per cluster were determined using the ReactomePA package^45^, with the Reactome pathway database (https://reactome.org/). First, gene symbols of the upregulated genes were converted to Entrez IDs, then pathways enriched at adjusted p-value < 0.05 were calculated.

### FACS

Single cell suspensions were stained with one of the three antibody panels as depicted in Supplementary Table 4. Briefly, cells were centrifuged at 300 g for 5 min and then resuspended in 100 μL PBS supplemented with 2% FCS. Next, cells were stained for 30 min at 4 °C, after which they were washed twice and resuspended in 400 μL PBS supplemented with 2% FCS. FACS data was generated using the BD LSR-II system (BD Bioscience) and analyzed using FlowJo v10 following the gating strategy as shown in Suppl. Figure 9.

#### Cell viability on FACS

Cells were stained using viability dye as depicted in Supplementary Table 4. Dead cells were determined by gating strategy as illustrated in Suppl. Figure 9.

#### Functional experiments

*Organoids* were generated using cells dissociated with the one-step collagenase protocol. The cells which were left after loading the 10 × chip for scRNA-seq were used for organoid culture. The cells were washed with 10 ml HGF-medium. Cells were centrifuged for 5 min at 1000 rpm at 4 °C, and supernatant was removed. 15ul of matrigel was added to the pellet, which was plated in a 96 wells plate. The plate was incubated 15 min upside-down in a 37 °C incubator. 200ul EM medium with primocin was added (1:500) to the wells. Organoids were expanded until the desired density for freezing in liquid nitrogen was achieved.

*IEL cells* (CD45 + CD3 + TCRαβ + CD8αβ + CD103 +) were isolated from gut tissue and expanded in vitro following protocol^46^. Briefly, dissociated cells from gut with IEL phenotype were sorted on a Beckman Coulter MoFlo Astrios cell sorter. IELs were cultured in RPMI with 10% Human AB serum (Sigma Aldrich, H4522), 0.1% IL-2 (RD System, AE5916003), 0.1% pPHA-L (Sigma, L2769-2MG) and a mix of feeder cells that consisted of irradiated PBMCs and Epstein-Barr virus transformed B cells from 3 different donors. Feeder cell mixes were prepared in a ratio of 10 PBMCs:1 EBV:1 CD8 + cell. Cells were then propagated during 10 days and analysed by FACS to confirm the purity of cells.

### Ethics statement

Biological material was obtained from patients according to protocols approved by the regional ethics committees (Medical Ethical Committee of University Medical Center Groningen and East of England, Cambridge South Research Ethics Committee), and the individuals donating material gave their written informed consent. The experimental protocols were approved by Medical Ethical Committee of University Medical Center Groningen (NL58808.042.16, NL24572.018.08) and East of England, Cambridge South Research Ethics Committee (17/EE/0338).

## Supplementary Information

Supplementary Information.

Supplementary Table 1.

Supplementary Table 2.

Supplementary Table 3a.

Supplementary Table 3b.

Supplementary Table 4a.

Supplementary Table 4b.

Supplementary Table 4c.

Supplementary Table 5.

Supplementary Table 6.

Supplementary Figures.

## Data Availability

The datasets generated during and/or analysed during the current study are available in the MOLGENIS^47^ repository, https://downloads.molgeniscloud.org/downloads/singelcell_methodspaper/. The FACS files supporting this study are available upon request to the authors.

## Code availability

All code is available through GitHub: https://github.com/WeersmaLabIBD/SingleCell/tree/master/Method_comparisons.
