# Supplementary material for: Gut mucosa dissociation protocols influence cell type proportions and single-cell gene expression levels
Source: Sci Rep. 2022 Jun 14;12:9897. doi: 10.1038/s41598-022-13812-y (PMC9197976; doi:10.1038/s41598-022-13812-y)
Supplement: Supplementary file 1 — Supplementary Information. [file 41598_2022_13812_MOESM1_ESM.docx]

Supplementary Information

**Gut mucosa dissociation protocols influence cell type proportions and single-cell gene expression levels**

Werna T.C. Uniken Venema^1,#^, Aarón D. Ramírez-Sánchez^2,#^, Emilia V. Bigaeva^1^, Sebo Withoff^2^, Iris Jonkers^2^, Rebecca E. McIntyre^3^, M. Ghouraba^3^, Tim Raine^4^, Rinse K. Weersma^2^, Lude Franke^1^, Eleonora A.M. Festen^1,*^, Monique G.P. van der Wijst^2,*^

^#^ shared first author, * corresponding authors

^1^ University of Groningen, University Medical Center Groningen, Department of Gastroenterology and Hepatology, Groningen, The Netherlands

^2^ University of Groningen, University Medical Center Groningen, Department of Genetics, Groningen, the Netherlands.

^3^ Wellcome Trust Sanger Institute, Wellcome Trust Genome Campus, Hinxton, UK.

^4^ Division of Gastroenterology, Department of Medicine, Addenbrooke's Hospital, University of Cambridge, Cambridge, UK.

**Supplementary Table 1**

Sample info sheet. Includes sample names and viability numbers, as measured through Trypan Blue staining or FACS stain. Used panels for FACS analysis are described in Supplementary Table 4.

**Supplementary Table 2**

Sequencing quality control parameters, as deriving from CellRanger demultiplexing software for the paired samples of the cryopreservation dataset and for the three different dissociation methods. Medians and standard deviations of the parameters are described. If known, CellRanger version and human genome reference version are described.

**Supplementary Table 3**

Number of differentially expressed (DE) genes for 1-step collagenase dissociated samples.

**A**: Differentially expressed genes per cell type for the 2-step collagenase vs 1-step collagenase protocols. **B**: Differentially expressed genes per cell type for the 3-step protease vs 1-step collagenase protocols.

**Supplementary Table 4**

Number of differentially expressed (DE) pathways upon each protocol and the influence of cryopreservation on these pathways.

**A:** 1-step collagenase versus 2-step collagenase protocol. **B**: 1-step collagenase vs 3-step protease. **C:** Overview of all comparisons per cell type

**Supplementary Table 5**

FACS panels for analyses of FACS data.

**Supplementary Table 6**

P-values after Wilcoxon rank sum test with Holm’s correction showing difference in cell type proportions between protocols, depicted in Figure 4s.

**Supplementary Figure 1. Parameters of quality control in scRNAseq after cryopreservation in one-step collagenase protocol**

To visualize sequencing quality between fresh (A) and cryopreserved (B) biopsies, number of UMI and number of Genes per cell were plotted. Since mitochondrial gene expression may indicate worse cell quality or stress, mitochondrial gene expression was visualized in relation to number of UMI per cell for C) freshly dissociated and B) cryopreserved gut mucosal cells. For distinction of major cell types, cells were colored by: immune (green), epithelial (red) or stromal (blue) category. E) Violin plot that shows the percentage of mitochondrial genes per cell type, showing bimodal distribution for some cell types. Cryopreserved cells in blue, freshly dissociated ones in green. Median of the percentage of mitochondrial genes is depicted in the lower part of the plot per cell type and storage condition. nUMI=number of UMI, nGene=number of genes.

**Supplementary Figure 2. Comparison of main cell types recovered after cryopreservation by one-step collagenase protocol assessed by FACS.** Boxplots of cell proportions characterized by FACS separated by cell type. The identity of each cell type is noted in the top part of each plot (full description of the characterization of each cell type is described in Suppl. Fig. 9). Means were statistically tested using non-paired t-test. Significant differences are marked with a p-value. Cryopreserved cells in blue, freshly dissociated ones in green. Sample size is depicted in the legend of the figure.

**Supplementary Figure 3. Parameters of quality control in scRNAseq per cell-dissociation protocol.**

Cell type quality parameters as obtained through scRNAseq are visualized by number of UMI versus number of genes expressed, for each of the described protocols. For distinction of major cell types, cells were colored by: immune (green), epithelial (red) or stromal (blue) category. nUMI=number of UMI, nGene=number of genes, F2=fraction 2.

**Supplementary Figure 4. Differences in cell type proportions recovered in each protocol by FACS.**

Boxplots of cell percentages (%) from total alive cells per cell type, determined by FACS analysis (full description of the characterization of each cell type is described in Suppl. Fig. 9). Compartments and dissociation protocols are indicated in the x-axis. Sample size is depicted in the legend of the plot. WB, whole biopsy; EL, epithelial layer; LP, lamina propria; F1, fraction 1; F2, fraction 2.

**Supplementary Figure 5. Differential expressed genes in epithelial cells from epithelial layer versus lamina propria in the two-step collagenase protocol.**

Volcano plot showing all 222 differentially expressed genes between epithelial cells derived from the collagenase-treated lamina propria layer versus the EDTA treated epithelial layer (adjusted p-value < 0.05, absolute L2FC > 0.25). In red the upregulated genes in the lamina propria, in blue the downregulated ones. 34 genes are labeled: these are known collagenase-induced genes.

**Supplementary Figure 6. Recovery of CD8 T cells and IELs is different between protocols.** Using CD8, CD56 (associated with cytotoxicity), and CD103 (found in 90% of IEL population) cell surface markers, the T cell population is characterized in-depth. We observe minor differences on the A) CD8+ cells recovered from the T cell population. However the proportion of CD103+ cells B) are reduced in the one-step protocol, thereby indicating a mixture of IELs and other CD8+ subpopulations. C) CD56+ marker expression suggests that the two-step protease protocol recovers more cytotoxic CD8+ cells, presumably the IEL phenotype, while the one-step protocol shows less CD8+cells, supporting a heterogenous CD8+ population. In D) representative FACS gating plots of CD8+ population in one-step collagenase (n=3) and E) two-step protease protocols, epithelial layer (n=3) and F) fraction 1 (n=3). The numbers in red indicate the percentage of each gated population. WB, whole biopsy; EL, epithelial layer; IF, immune fraction.

**Supplementary Figure 7. Differentially expressed genes between epithelial cells and B cells isolated with different protocols.** Heatmaps of 10 upregulated genes between protocols for A) epithelial cells and B) B cells. 1-coll WB=one-step collagenase whole biopsy, 2-coll EL= two-step collagenase epithelial layer, LP = lamina propria layer, 3-prot=three-step protease, epi=epithelial layer

**Supplementary Figure 8. Functional experiments to grow organoids and expand IELs.** Organoids were generated using the cells dissociated with the one-step collagenase protocol. In A), we show a representative picture of the organoids at day 7 after starting the expansion. IEL cells (CD45+ CD3+ TCRαβ+ CD8αβ+ CD103+) were isolated from gut tissue and expanded in vitro. In B) we show a representative picture of the IELs at day 7. After day 10, cells were analyzed by C) FACS to confirm the purity of cells.

**Supplementary Figure 9. Gating strategy used to identify cells by FACS.** Gating strategy followed to characterize the cells obtained after the dissociation in the different protocols and preservation conditions. Due to the use of different antibody panels, we followed a gating strategy that allows us to obtain the same cell phenotypes regardless of the antibody composition. In red are the phenotype names used for each population.

**Supplementary Figure 10. Gene markers specific for cell types.** Violin plots of expression levels of marker genes showing that cell type prediction based on ‘scPred’ prediction produces reliable results.
