# Supplementary material for: Gut mucosa dissociation protocols influence cell type proportions and single-cell gene expression levels
Source: Sci Rep. 2022 Jun 14;12:9897. doi: 10.1038/s41598-022-13812-y (PMC9197976; doi:10.1038/s41598-022-13812-y)

Gut mucosa dissociation protocols influence cell type proportions and single-cell gene expression levels

Werna T.C. Uniken Venema[1,#], Aarón D. Ramírez-Sánchez[2,#], Emilia V. Bigaeva[1], Sebo Withoff[2], Iris Jonkers[2], Rebecca E. McIntyre[X], M. Ghouraba[X], Rinse K. Weersma[2], Lude Franke[1], Eleonora A.M. Festen[1,*], Monique G.P. van der Wijst[2,*]

[#] shared first author, * corresponding authors

[1] University of Groningen, University Medical Center Groningen, Department of Gastroenterology and Hepatology, Groningen, The Netherlands

[2] University of Groningen, University Medical Center Groningen, Department of Genetics, Groningen, the Netherlands.

Supplementary Figure 1. Parameters of quality control in scRNAseq after cryopreservation in one-step collagenase protocol

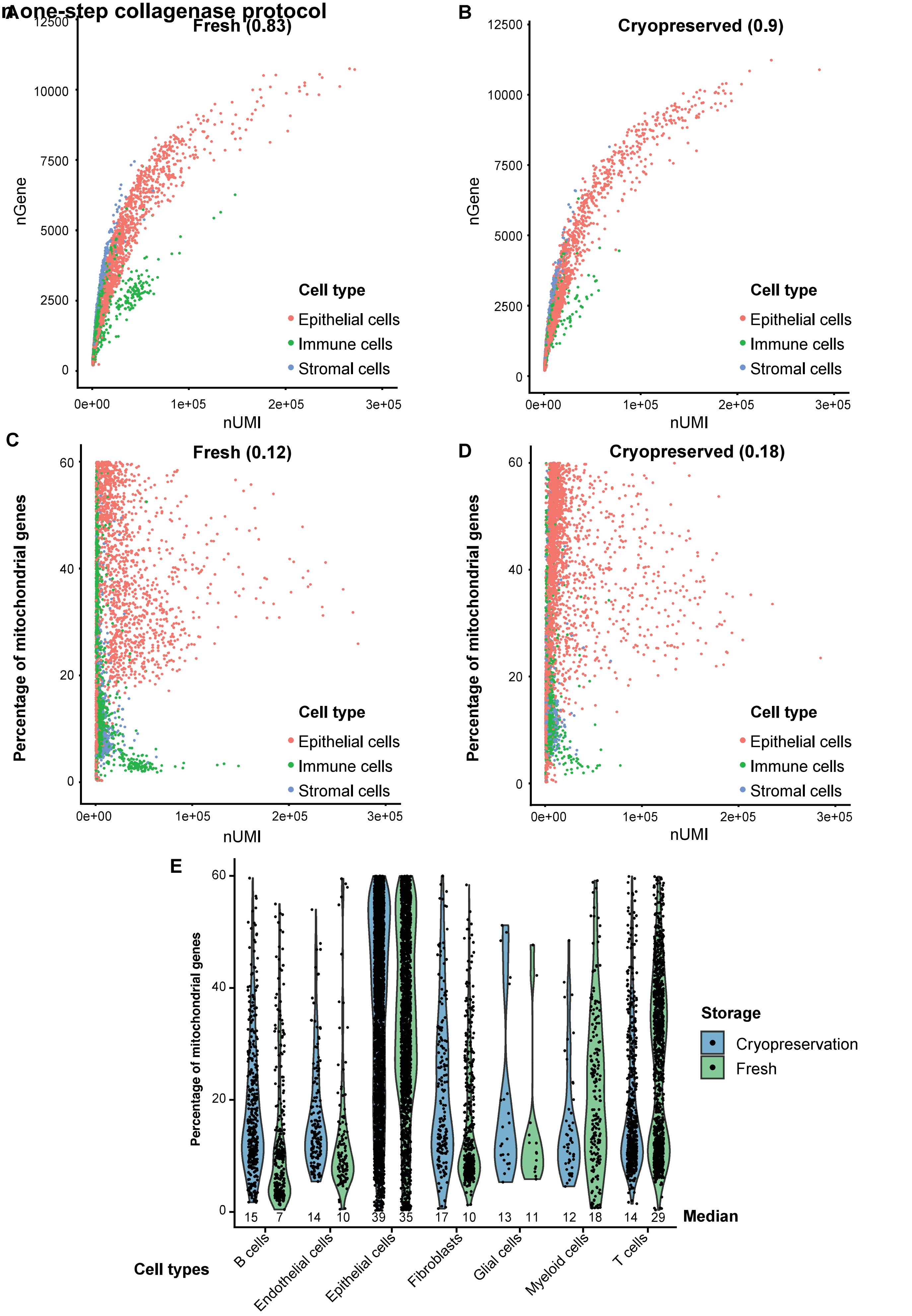

Supplementary Figure 2. Comparison of main cell types recovered after cryopreservation by one-step collagenase protocol assessed by FACS

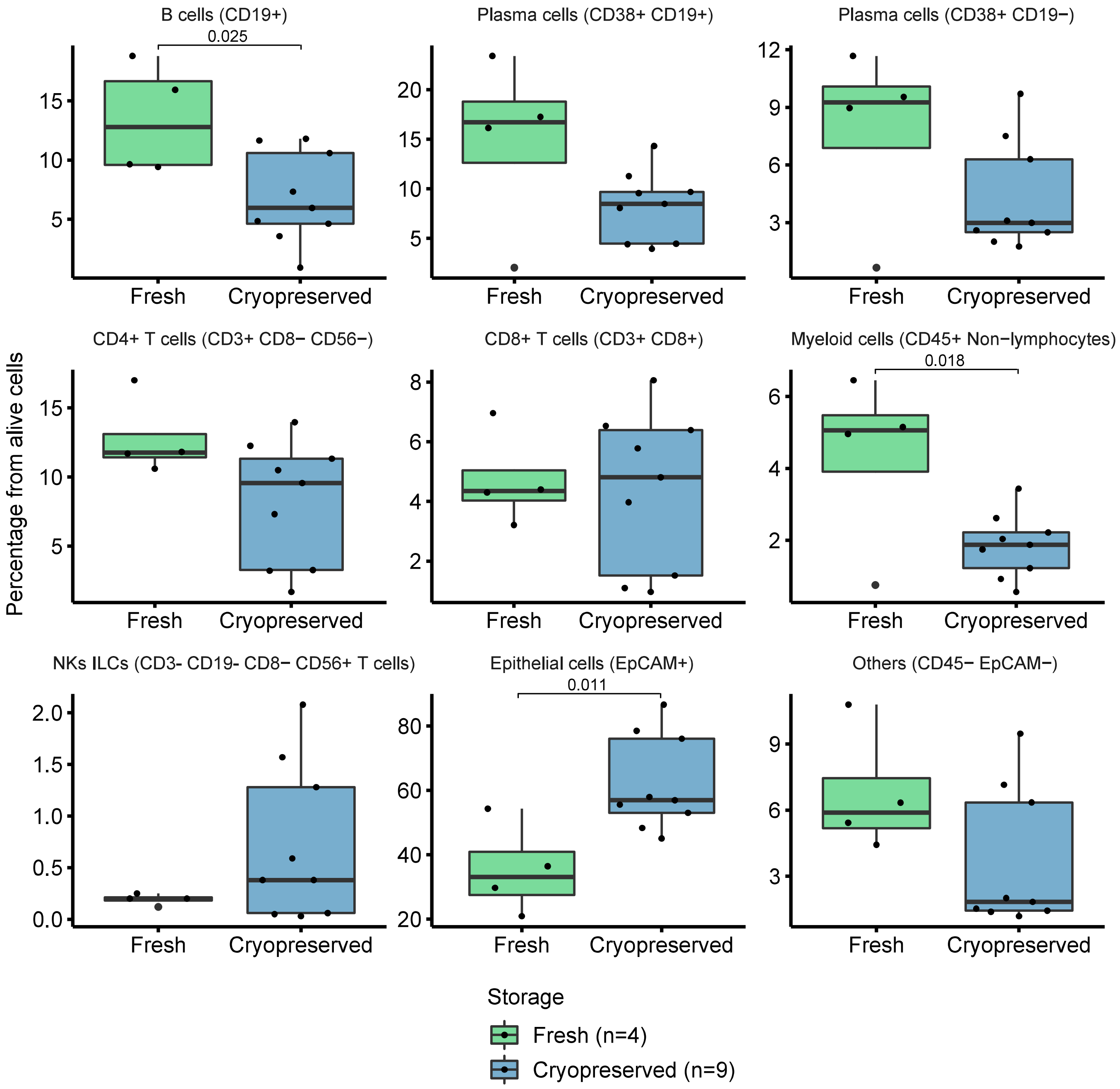

Supplementary Figure 3. Parameters of quality control in scRNAseq per cell-dissociation protocol

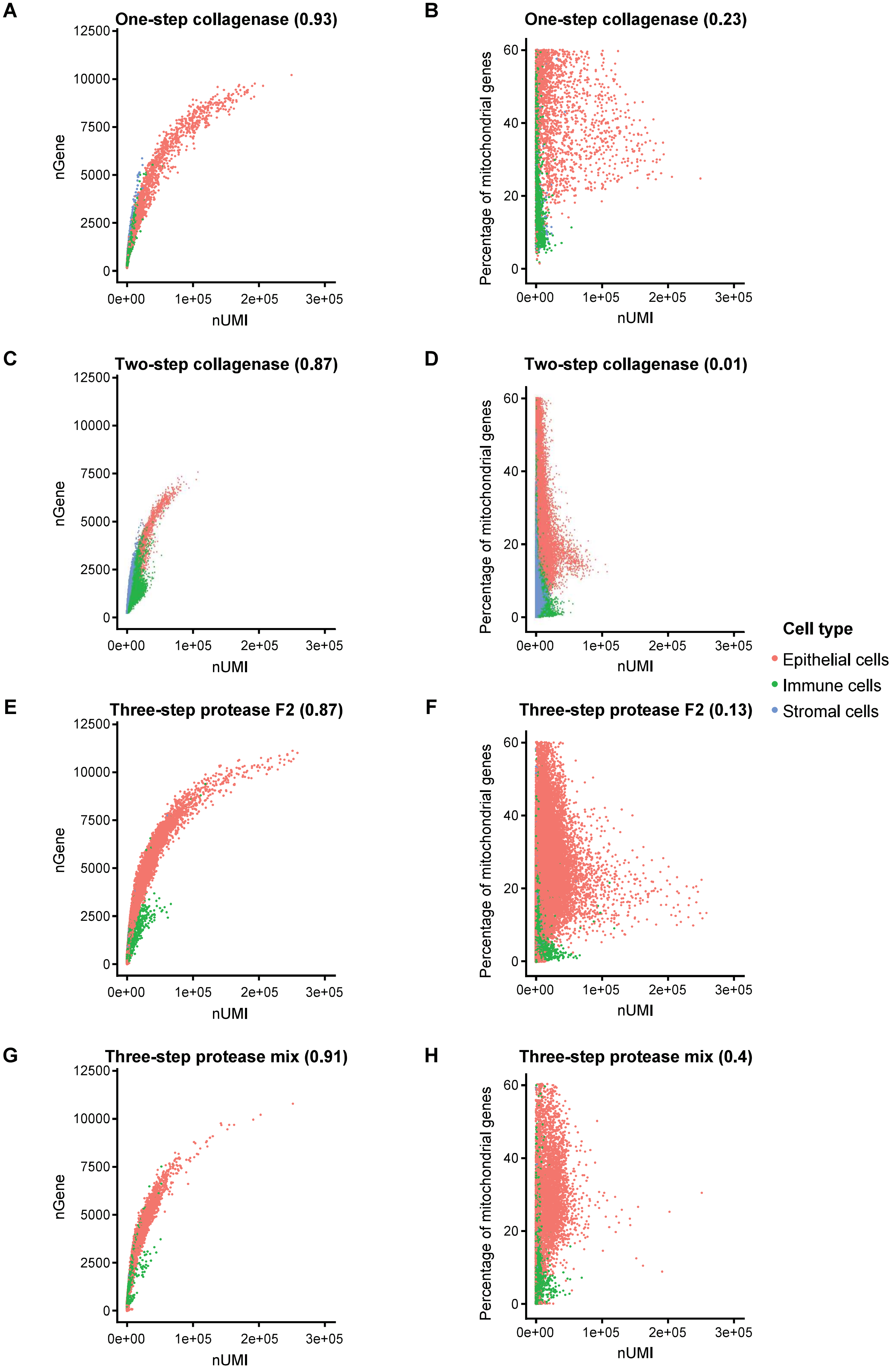

Supplementary Figure 4. Differences in cell type proportions recovered in each protocol by FACS

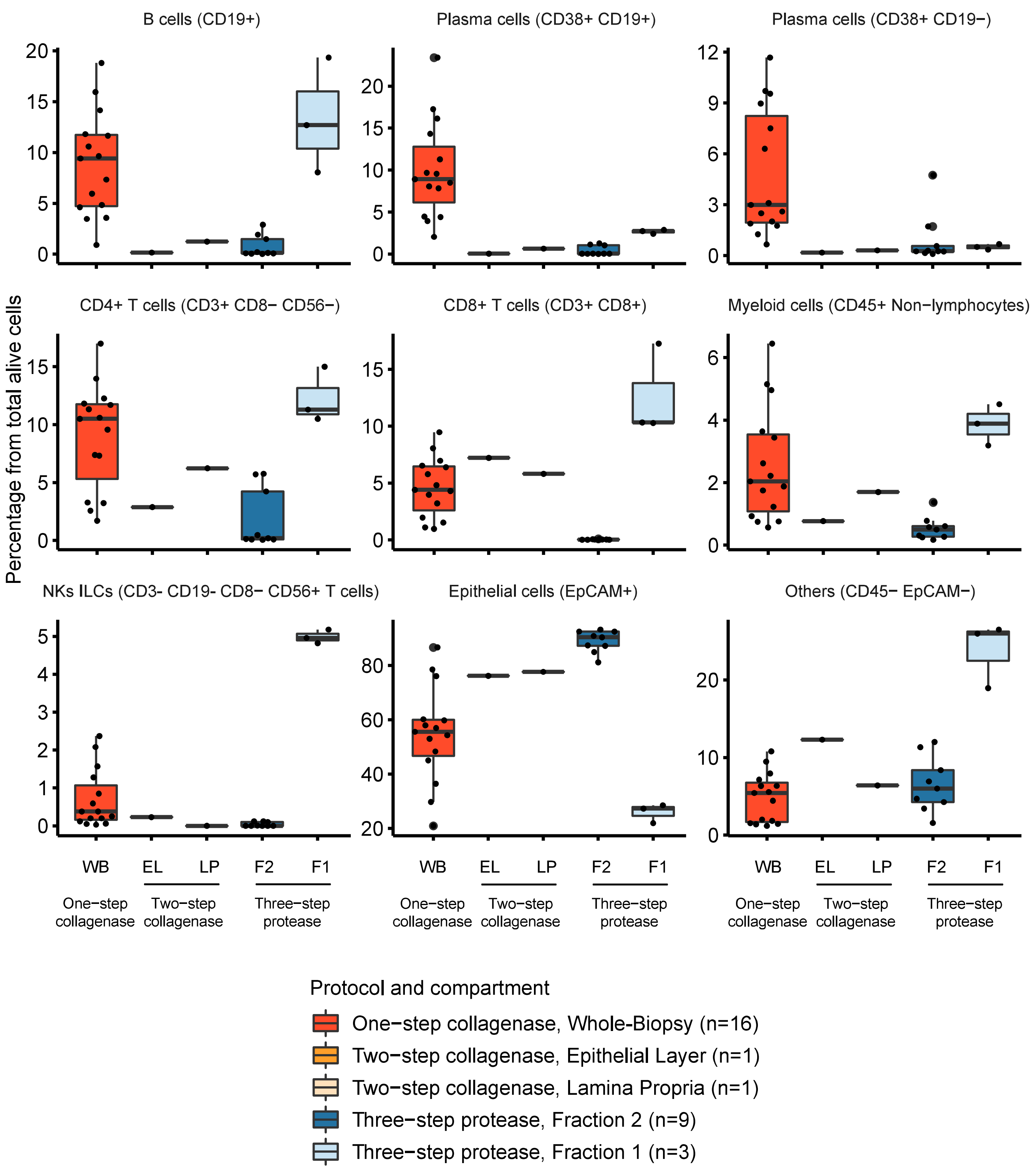

Supplementary Figure 5. Differential expressed genes in epithelial cells from epithelial layer versus lamina propria in the two-step collagenase protocol

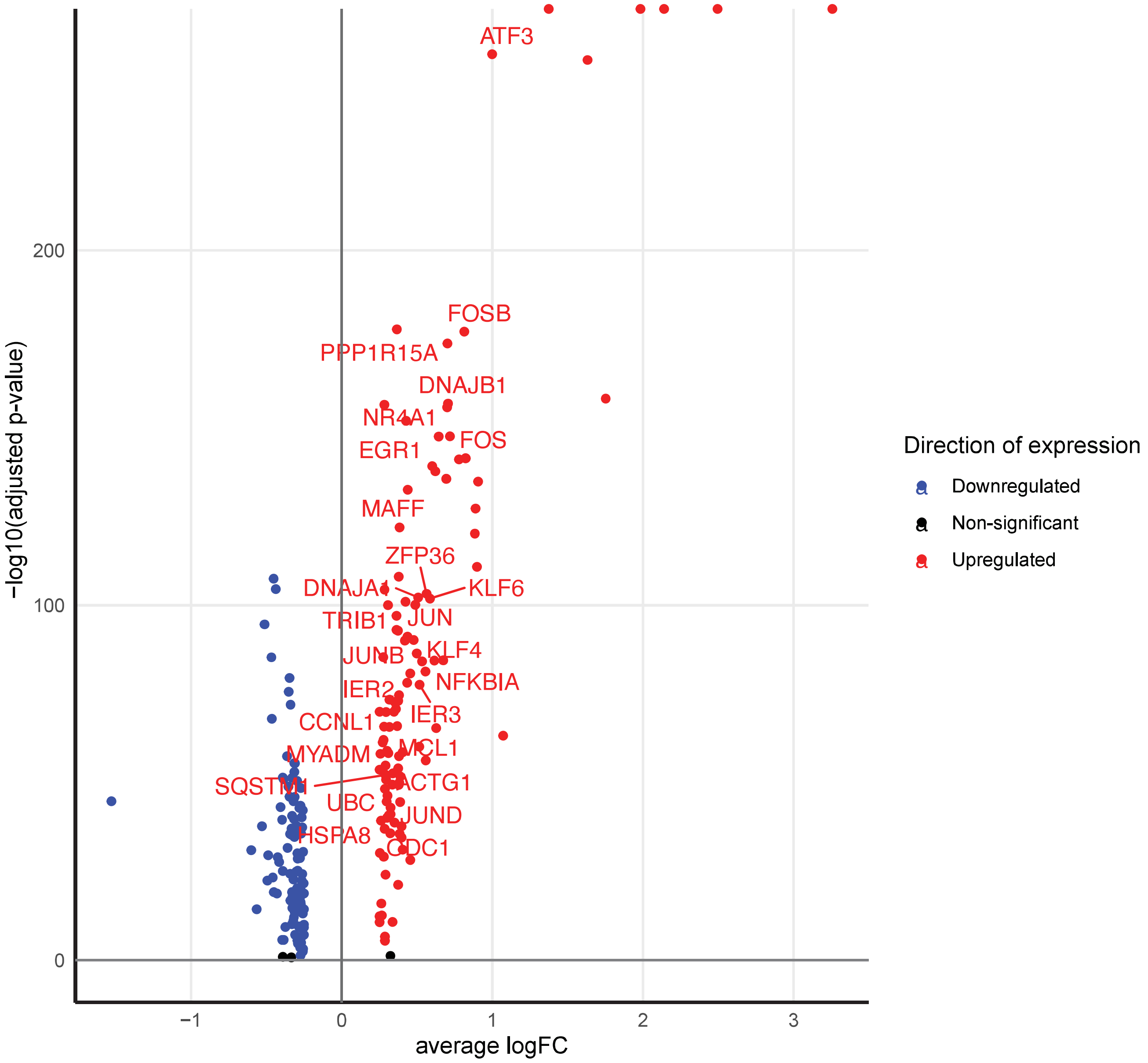

Supplementary Figure 6. Recovery of CD8 T cells and IELs is different between protocols

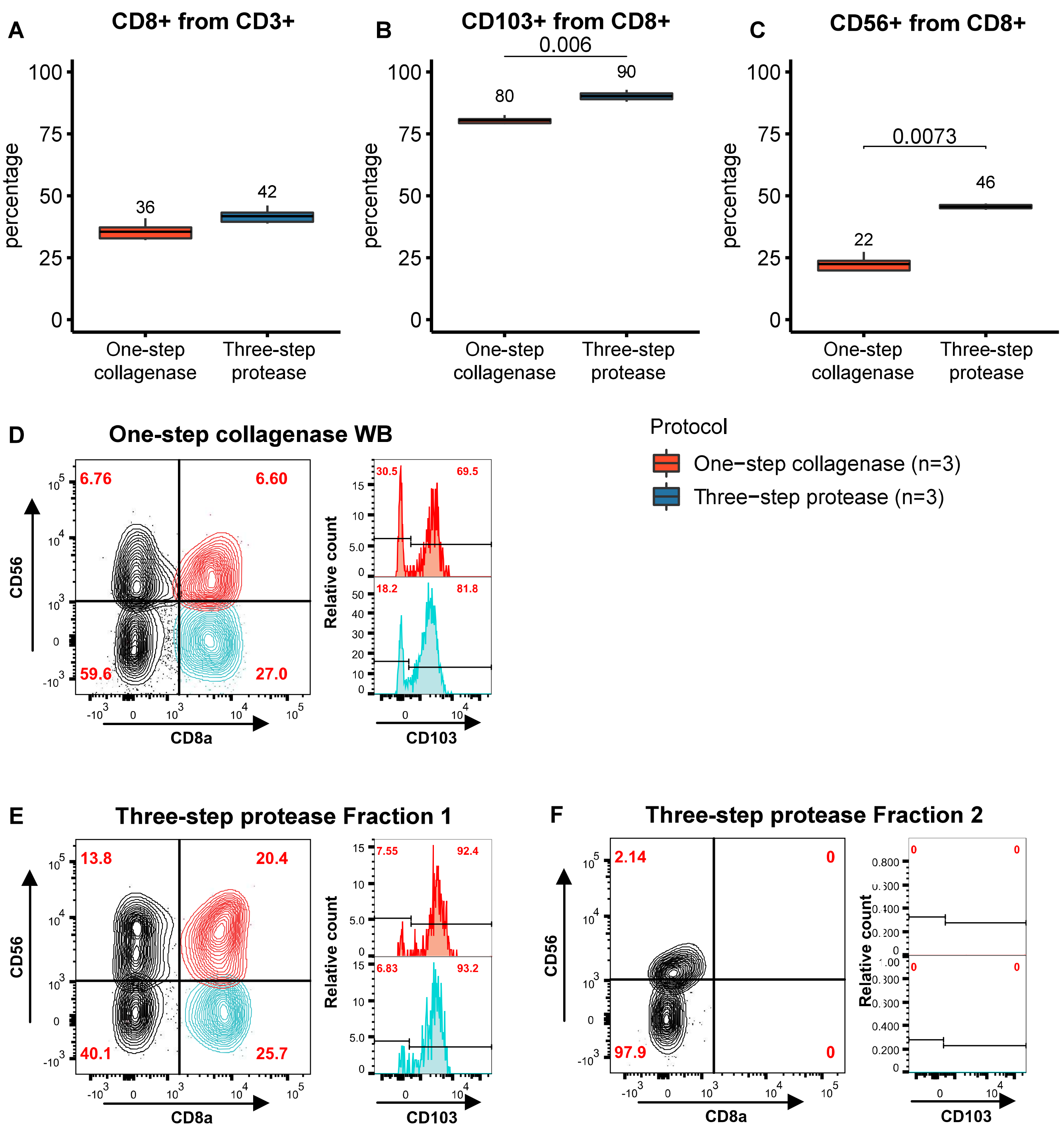

Supplementary Figure 7. Differentially expressed genes between epithelial cells and B cells isolated with different protocols

A

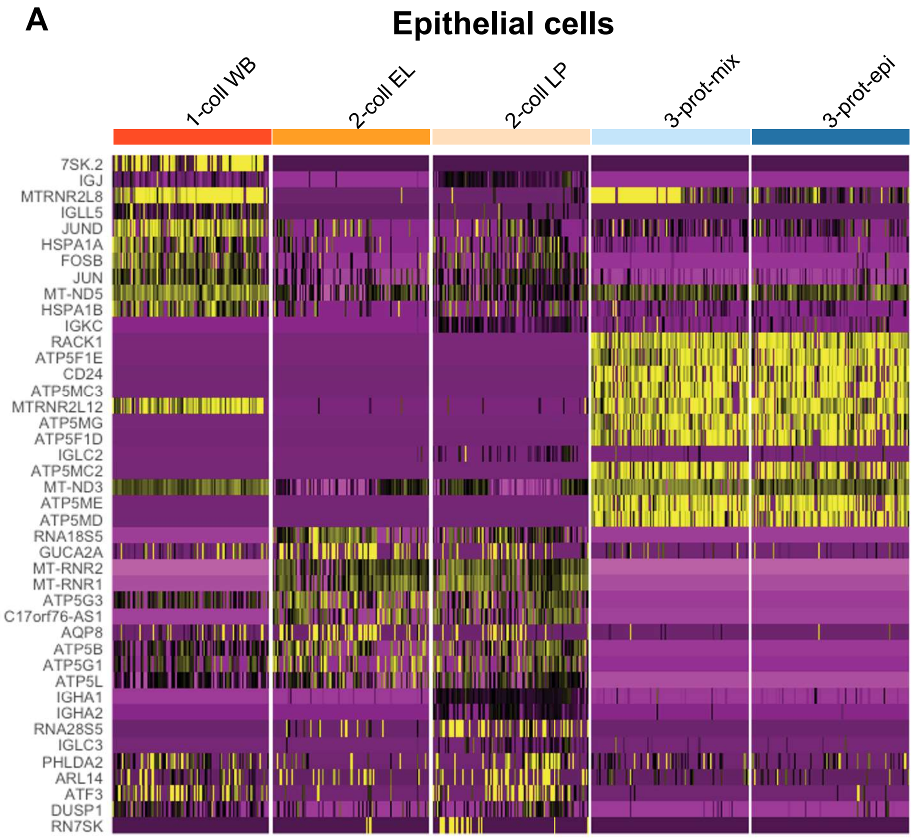

B

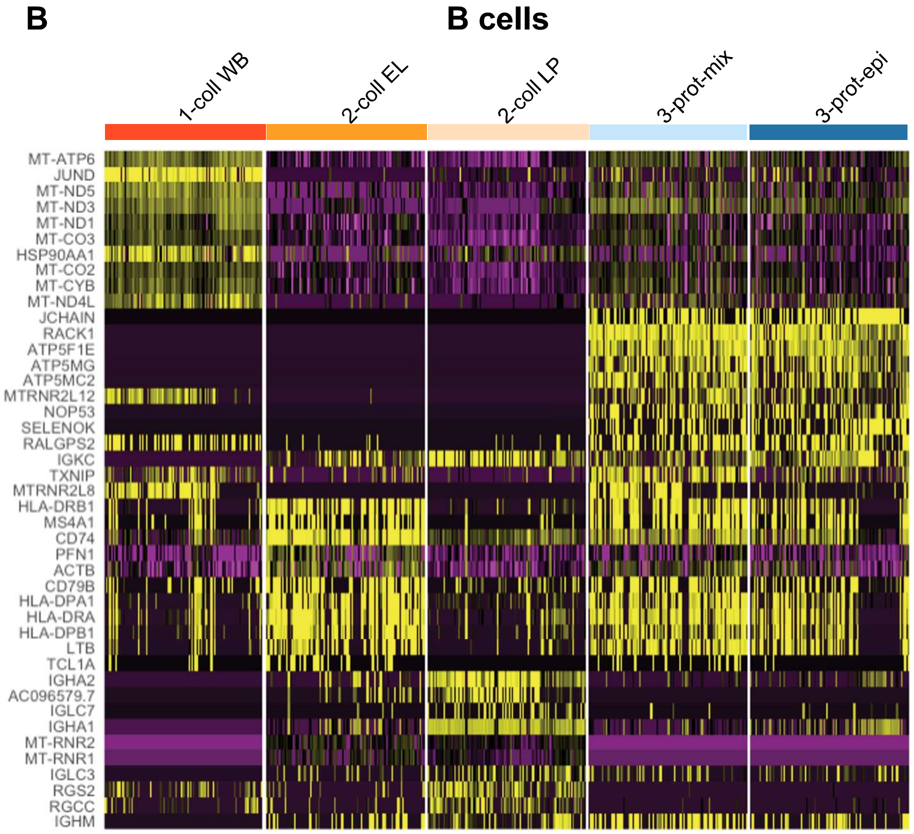

Supplementary Figure 8. Functional experiments to grow organoids and expand IELs.

A

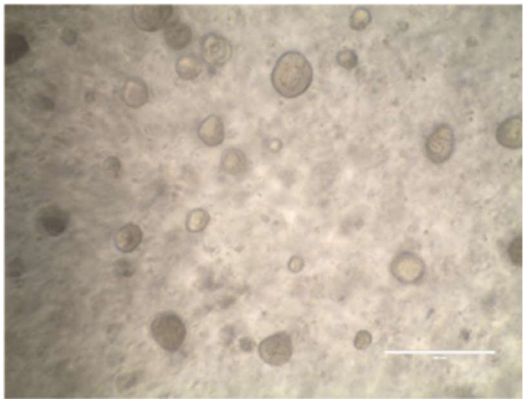

B

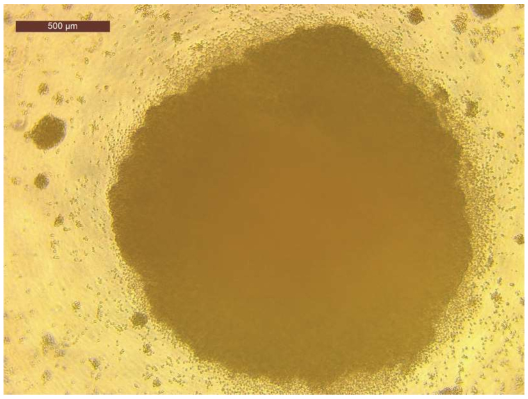

C

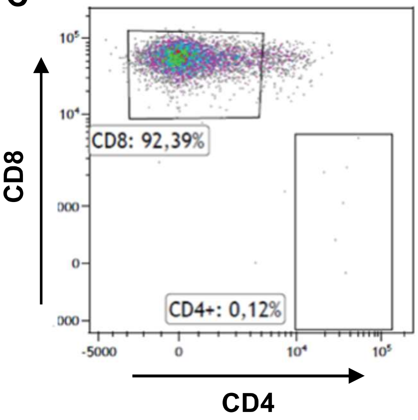

Supplementary Figure 9. Gating strategy used to identify cells by FACS.

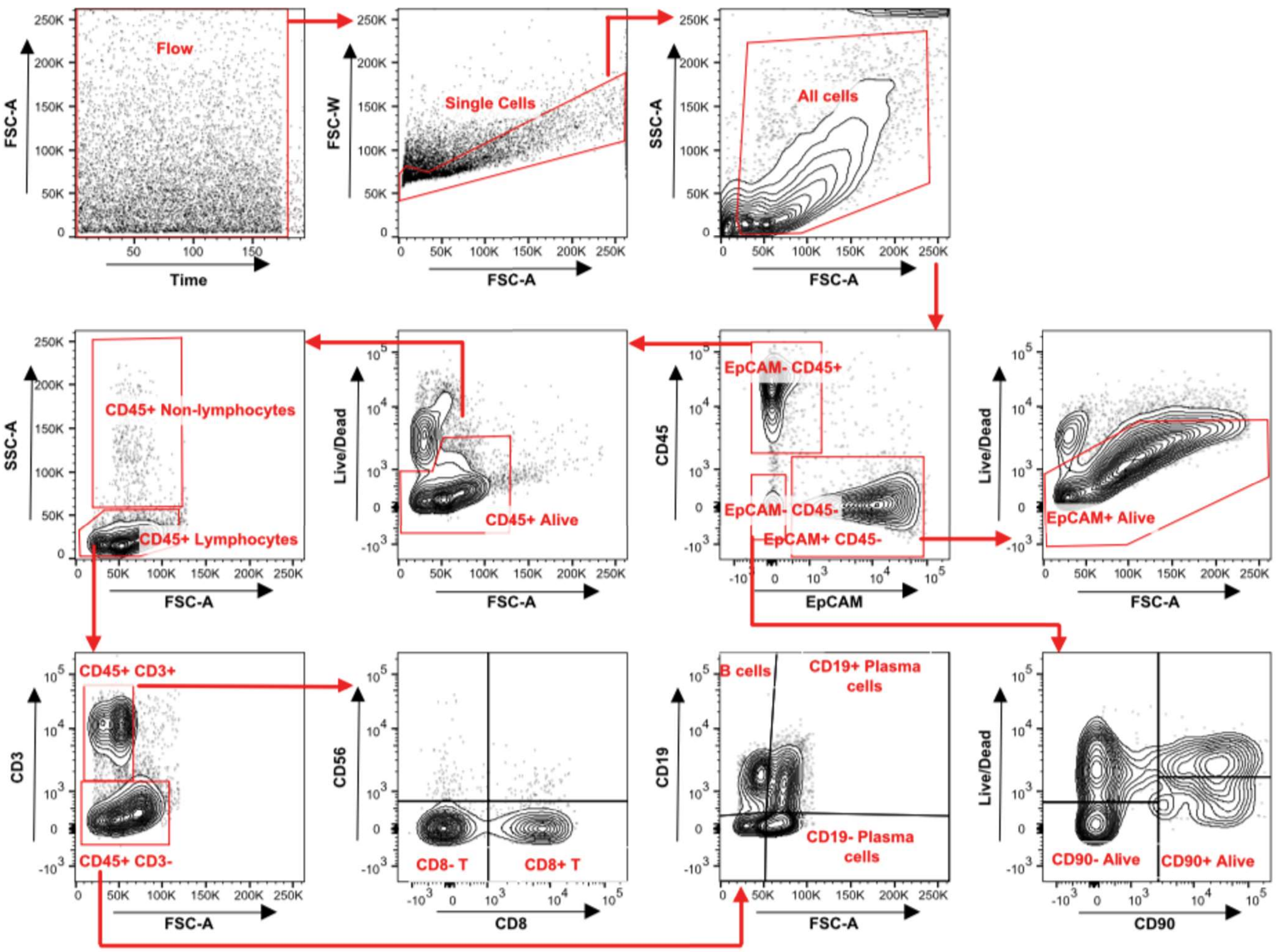

Supplementary Figure 10. Gene markers specific for cell types

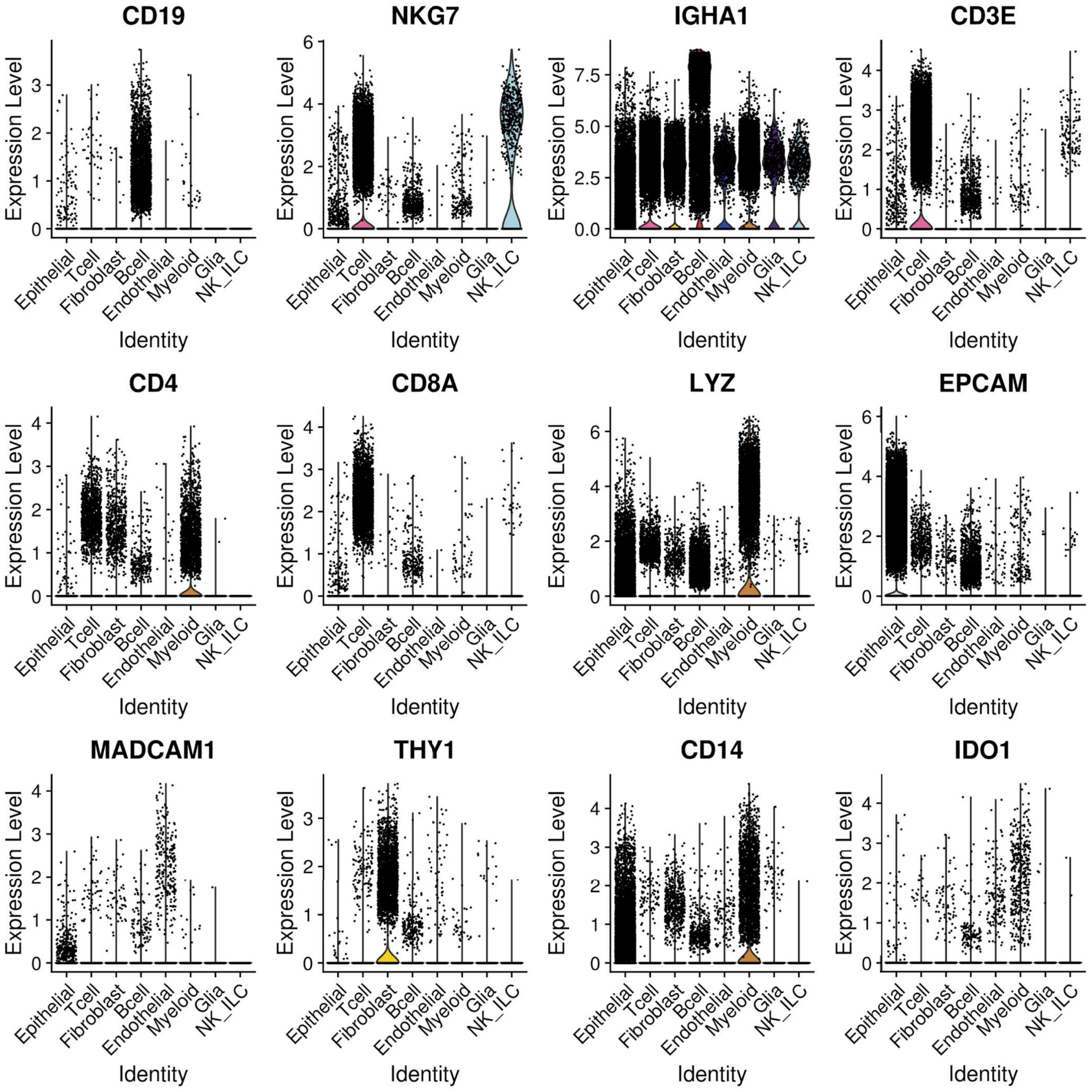

Supplement: Supplementary file 11 — Supplementary Figures. [file 41598_2022_13812_MOESM11_ESM.pdf]
